# Analectic

**Published:** 1828-05

**Authors:** 


### MISCELLANEOUS INTELLIGENCE.

---

### I. ANALECTIC.

1. *Permanent Evidence of Successful Vaccination.* Under this head, Dr. George Gregory has published a paper in the MAY No. of the Medical and Physical, in which he modestly disclaims all title to much novelty—and only hopes to be useful, by refreshing the memory on certain minute points connected with vaccination. We shall give a very brief abstract of these minute points.

First. A proper vaccine scar should be distinctly defined, even after a lapse of 20 years; in order to which, it is nearly indispensable that the scab should remain on—or, at least, that cicatrization should not be completed till the 21st day. In some cases, the cicatrix is formed by the 14th or 15th day—and then "vaccination is imperfect."

Second. The true and perfect vaccine scar is circular, or nearly so. When common inflammation supervenes early, the scar is irregular in form, and the system is still open to the small-pox, more or less modified. The diameter of the circular scar is not material. The largest, however, which he considers compatible with safety, will be that of a six-pence, or small wafer.

Third. The vaccine scar should be indented and radiated; though he does not insist on these appearances as a *sine qua non* in the proofs of perfect vaccination.

The sources of imperfection in the process of vaccination are chiefly the following:—effete virus; hence the inocculation should always be with fresh matter, and not by points, if possible:—pre-occupation of the system by some other important process, as dentition, visceral inflammation, fever, hooping-cough, porrigo favosa, or herpes:—and, lastly, a too advanced period of life at the time the process of vaccination is instituted.

For several collateral remarks and observations, all of a practical nature, and all indicative of the sound judgment and accurate discrimination by which the author is characterised, we must refer to the original paper in our cotemporary for May last.

In the same journal there are two papers connected with the subject of vaccination—one by Mr. Dalton, an eminent surgeon of Bury St. Edmonds, who describes an epidemic variola, against which vaccination made a bold and successful stand—the other by Dr. Morton, Physician to the Metropolitan Infirmary for Children, in which it is shown that, in his own experience at least, the proportion of variolous attacks after vaccination does not exceed four per cent. at the most. In the case of failure, the disease was generally very mild." *Med. Chirur. Rev. Oct.* 1827, *p.* 459.

2. *Ununited Fracture Cured by Pressure.* In a note to the article on "carotid aneurism" in our last number we alluded to this case but gave no particulars of the treatment. These have since been detailed by Mr. Brodie in our esteemed contemporary for July, and it may not be uninteresting, just to glance at the means by which the cure was affected.

J. M'Ewen, æt. 24, broke his right arm and left leg, in November, 1825. He was taken to a hospital and treated in the usual way.—The fracture of the leg did well, but no union took place in the arm. In August, 1826, he went to Panton Square, when Mr. Wardrop passed a seton, which was withdrawn at the end of a week. After a time the patient was discharged, and reported as *cured* in the Lancet. In Nov. 1826, he entered St. George's, the broken ends of the bone appearing to be united by ligament, riding one over the other, and admitting of extensive motion. Mr. Brodie now determined on applying pressure, on the principle suggested by Mr. Amesbury. The fore-arm being semi-bent, a wooden splint adapted to its figure, and reaching from the axilla to the fingers, was applied on the inside. On the outside of the arm, a straight splint was placed, extending from the shoulder to the outer condyle, and both splints were then secured by bandages. Over all there was a tourniquet, the band of which embraced the fracture, whilst the degree of pressure thus made on the broken bone was easily regulated by the screw, which was on the outside of the arm. The splint on the inside being broader than the limb, and only slightly concave, the principal vessels were defended from pressure, and whatever was the force employed, the circulation was but little interrupted. In six weeks, the motion of the fractured bones was much diminished, and at the end of three months none was perceptible. On the 21st May, the man left the hospital, the bones being firmly consolidated and the arm as useful as before the accident.

This case does credit to the ingenuity and perseverance of Mr. Brodie, and we have no doubt that pressure, properly and steadily applied, will prove effectual in many, if not most, of those cases of ununited fracture, which have been not unfrequently an approbrium to surgeons and surgery." *Ibid, p.* 478.

